# Mechanisms of Hierarchical Cortical Maturation

**DOI:** 10.3389/fncel.2017.00272

**Published:** 2017-09-11

**Authors:** Taylor Chomiak, Bin Hu

**Affiliations:** Division of Translational Neuroscience, Department of Clinical Neurosciences, Hotchkiss Brain Institute, Cumming School of Medicine, University of Calgary Calgary, AB, Canada

**Keywords:** hierarchical maturation, neocortex, development, maturation, pyramidal neuron

## Abstract

Cortical information processing is structurally and functionally organized into hierarchical pathways, with primary sensory cortical regions providing modality specific information and associative cortical regions playing a more integrative role. Historically, there has been debate as to whether primary cortical regions mature earlier than associative cortical regions, or whether both primary and associative cortical regions mature simultaneously. Identifying whether primary and associative cortical regions mature hierarchically or simultaneously will not only deepen our understanding of the mechanisms that regulate brain maturation, but it will also provide fundamental insight into aspects of adolescent behavior, learning, neurodevelopmental disorders and computational models of neural processing. This mini-review article summarizes the current evidence supporting the sequential and hierarchical nature of cortical maturation, and then proposes a new cellular model underlying this process. Finally, unresolved issues associated with hierarchical cortical maturation are also addressed.

## Introduction

The concept of cortical hierarchy has been widely recognized for years (Guillery, 2005). It is based on established structure-function relationships in the thalamo-cortical system that consist of primary sensory areas and several distinct higher-order association areas that are important for cognitive functions (Komura et al., 2001; Hu, 2003; Guillery, 2005; Shipp, 2007; Redgrave et al., 2010). Area-specific functions become more and more integrative as neural information moves through successive cortical tiers in the hierarchy. Historically, there has been debate as to whether postnatal cortical maturation of these hierarchies proceeds sequentially or simultaneously (Guillery, 2005). Whether the cortex matures sequentially or simultaneously has important implications. Answering this question is critical to our understanding of the basic neurobiological processes involved in brain maturation and cognitive function. It will also further our understanding of aspects related to adolescent behavior, neurodevelopmental disorders and emergent properties associated with neuro-computational models (Quartz, 1999; Guillery, 2005; Westermann et al., 2006; Aimone and Weick, 2013; Chomiak and Hu, 2013; Chan et al., 2016).

Much of the earlier work supporting simultaneous maturation was based on synaptic counts (Rakic et al., 1986; Bourgeois et al., 1994; Guillery, 2005). These classical publications supported the theory that synapse development occurs concurrently in anatomically and functionally diverse cortical regions. That is, these findings were in marked contrast to the traditional view of a hierarchical pattern of cortical development (Rakic et al., 1986). It is important to note that the evidence for hierarchical maturation largely stemmed from axonal myelination patterns (Guillery, 2005). Some of the earliest accounts of hierarchical cortical maturation came from Flechsig (1920). His work showed that pathways to the primary motor and sensory cortices are the first to myelinate, followed by adjacent cortical areas, and with delayed axonal myelination in higher-order cortical regions (Guillery, 2005). Similar synaptic count data also supported a hierarchical pattern of cortical maturation (Huttenlocher and Dabholkar, 1997; Guillery, 2005). However, it is important to note that these studies (that used synaptic counting methods to support either hierarchical or simultaneous maturation) have been questioned based on methodological limitations (Guillery, 2005; Elston and Fujita, 2014). For instance, in addition to limitations associated with synaptic identification (which depend on the orientation of tissue cutting), a synapse, which is large relative to section thickness, can be counted many times. Corrections for this well recognized error becomes more difficult with larger object size to section thickness ratios (Guillery, 2005). Thus, while there may appear to be more evidence to support a general view of hierarchical cortical development, the contradictory published synaptic count data, as pointed out by Guillery (2005), “left a disconcerting air of uncertainty about any possible sequence of maturation”.

Numerous cellular, behavioral and human brain imaging studies have subsequently been published since Guillery’s opinion article and most support the hypothesis of a hierarchical pattern of cortical maturation (Bourne and Rosa, 2006; Golarai et al., 2007; Hishida et al., 2007; Shaw et al., 2007; Sia and Bourne, 2008; Supekar et al., 2009; Asato et al., 2010; Bianchi et al., 2013; Ordaz et al., 2013; Shi et al., 2013; Elston and Fujita, 2014; Chan et al., 2016; Chomiak et al., 2016; Friedrichs-Maeder et al., 2017). In the following sections, we will summarize key findings from some of the human and animal studies that support a hierarchical cortical maturation model.

## Recent Brain Imaging Studies in Children

The question of how functional brain networks develop and mature in children is now a hot topic (Chan et al., 2016). This is because pathways that connect cortical regions important for cognitive functioning are also thought to exhibit a hierarchical pattern of maturation (Chan et al., 2016). Cortico-cortical pathways and cortico-subcortical loops serve as a conduit for top-down effects related to attention and other higher-order cognitive functions (Shipp, 2007). This is particularly evident when there is functional and/or structural pathology in these pathways and loops as this can lead to neurological dysfunction (Courchesne and Pierce, 2005; Minshew and Williams, 2007; Redgrave et al., 2010). Even cortico-subcortical loops are organized into structural and functional hierarchies, with primary sensory cortical regions exhibiting strong connectivity with lower-order subcortical sensory areas, and higher-order cortical regions exhibiting strong connectivity with higher-order subcortical areas (Komura et al., 2001; Hu, 2003; Shipp, 2007; Redgrave et al., 2010). Using a graph theory approach to examine developmental changes in large-scale cortical and subcortical functional organization, Supekar et al. (2009) were the first to investigate developmental changes in the functional organization of large-scale networks at the whole-brain level. They found that the brains of children had less hierarchical organization than those of young adults, characterized by reduced cortico-cortical functional connectivity and greater subcortical-primary sensory cortical connectivity. However, consistent with delayed functional connectivity between high-order cortical areas or successive cortical tiers (Shipp, 2007; Asato et al., 2010), increased cortico-cortical connectivity emerged with advancing age (Supekar et al., 2009). In fact, as pointed out by Supekar et al. (2009), greater cortico-cortical connectivity in young adults relative to children supports the view of increased myelination of axonal fiber tracks with advancing age as suggested by Flechsig (1920) almost a century ago.

In addition, Friedrichs-Maeder et al. (2017) also recently reported that cortical gray matter regions and associated white matter connections show corresponding gray and white matter maturation levels during early development. Based on these observations, this group also proposed a simple random-walk model to investigate possible mechanisms responsible for brain tissue maturation and the role of white matter connectivity (Friedrichs-Maeder et al., 2017). The computational model considered the movement of “signals” through each cortical region or white matter connection as an index of local maturation, with the probability of moving from one cortical region to another cortical region proportional to the weighted connectivity measured from tractography between the two regions (Friedrichs-Maeder et al., 2017). Their conclusion was that neural signals are relayed hierarchically through primary receiving cortical regions to higher-order cortical processing regions, and that the brain connectome may play an important role in propagating maturational signals (Friedrichs-Maeder et al., 2017). It remains to be determined what these maturational signals precisely are, and whether they simply serve to provide electrical activity, physical contact, or trophic support. Nevertheless, these results bolster the continually growing evidence in favor of hierarchical cortical maturation in humans (e.g., Gogtay et al., 2004; Golarai et al., 2007; Shaw et al., 2007; Supekar et al., 2009; Asato et al., 2010; Ordaz et al., 2013; Chan et al., 2016; Friedrichs-Maeder et al., 2017).

## Recent Cellular and Network Studies in Animals

**Figure 1:**
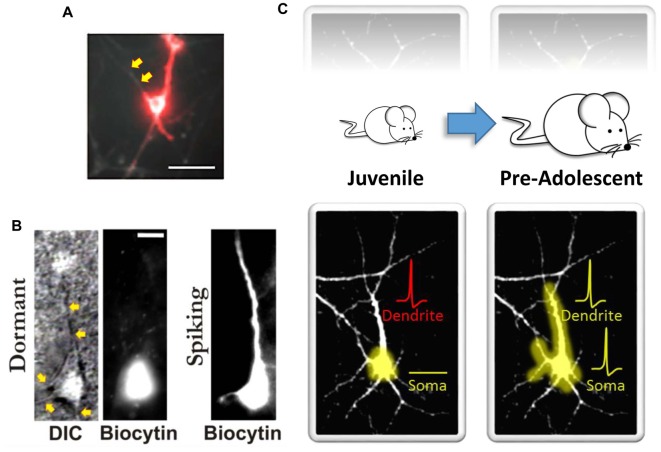
Somato-dendritic decoupling in neurons. **(A)** Optical imaging using microbial rhodopsin in an immature (10–14 days *in vitro*) hippocampal neuron. Red indicates an action potential. As noted by the authors, “the process extending to the top left of the cell body does not appear in the red channel; it is electrically decoupled from the cell” (indicated here by the yellow arrows). Panel **(A)** adapted by permission from Macmillan Publishers Ltd: Nature Methods (Kralj et al., 2011), copyright (2011) http://www.nature.com/naturemethods/. **(B)** Identified high-order temporal lobe neocortical dormant neurons (*left*) from Chomiak et al. (2016) that exhibit somato-dendritic decoupling. Yellow arrows indicate observable dendrites that lack biocytin labeling. Biocytin was delivered via patch pipette during patch-clamp recordings to electrophysiologically confirm a non-excitable and functionally compartmentalized soma (not shown here). Spiking neurons (*right*) exhibit somato-dendritic coupling; dendritic biocytin dye labeling and associated membrane capacitance confirmation. **(C)** A schematic illustrating that the development of somato-dendritic coupling (*bottom*) in the high-order temporal lobe is protracted (*top*), with a greater proportion of neurons in the juvenile stage exhibiting decoupling. Here dendrites can receive afferent inputs and even spike (denoted in red), but this information does not converge at the level of the soma. This may help keep recurrent connections “off-line” during postnatal development. Panel **(B)** taken, and Panel **(C)** modified, from Chomiak et al. (2016); Springer Nature (2016) © Chomiak et al. (2016) Open Access. This article is distributed under the terms of the Creative Commons Attribution 4.0 International License (http://creativecommons.org/licenses/by/4.0/).

Although human brain imaging studies have provided convincing new evidence for hierarchical cortical maturation, the results offer only limited insight into the functional and structural constraints that make such a hierarchical pattern of cortical maturation possible. In contrast, remarkable progress has been made in this regard using various experimental approaches in animal models. For example, similar to earlier findings in non-human primate indicating that the maturation of functional connectivity proceeds in a hierarchical fashion (Zhang et al., 2005; Kourtzi et al., 2006), Hishida et al. (2007) reported long-lasting maturation of functional connectivity from higher-order association cortical regions to lower-order primary sensory cortical regions. Using an *in vitro* rat preparation coupled with activity-dependent changes in endogenous fluorescence derived from glutamatergic transmission, they found that functional feedback connections from higher-order temporal lobe association areas were quite weak compared to the feedforward connections arising from the primary auditory cortex (A1; Hishida et al., 2007). It was also shown that unlike A1 which has an early postnatal developmental critical period for feedforward functional activity (i.e., primary cortical area → association cortical area), a relatively long postnatal critical period exists for the development of functional feedback connectivity from higher-order regions (i.e., association cortical area → primary cortical area; Hishida et al., 2007). Interestingly, stimulation of gray matter in association areas did result in some local depolarization. However, surprisingly, it largely failed to transmit back to the lower-order A1 region (Hishida et al., 2007). What may explain these findings? One possibility is that neurons in the slower maturing higher-order cortical areas possess immature dendritic arbors with fewer excitatory synapses on dendritic spines. This could lead to reduced excitatory drive and postsynaptic neuronal depolarization of these feedback pathways. For example, we recently evaluated GFP-transfected single-cell morphological developmental trajectories of higher-order association cortical neurons and compared them to other brain regions over the first several weeks of postnatal neuronal development *in vitro*. We evaluated several structural features often used as an index of neural maturity (Quartz and Sejnowski, 1997; Guillery, 2005). Unlike hippocampal or primary sensory cortical neurons, higher-order temporal lobe association neurons exhibited reduced dendritic growth trajectories (Chomiak et al., 2016). In addition, reduced dendritic spine density and increased immature-type dendritic spines were more common in neurons in association cortex compared to primary sensory cortex (Chomiak et al., 2016). However, as noted above with the work of Hishida et al. (2007), association cortical neurons do appear to receive excitatory input from primary sensory cortical regions. This therefore suggests a discordance between inputs onto dendrites and output signaling via the soma. In fact, this concept was recently demonstrated *in vivo* where Moore et al. (2017) showed a poor correlation of activity between dendritic and somatic compartments. Furthermore, optical recording of action potentials using microbial rhodopsin has shown that dendritic branches can be electrically decoupled from the soma (Kralj et al., 2011; Figure 1A), and computational models have also supported the notion that increasing intra-dendritic resistance can lead to decoupling of dendritic and somatic compartments and influence synaptic electrophysiology and the emergence of mature electrophysiological firing patterns (Mainen and Sejnowski, 1996; Bekkers, 2011). Together, these observations suggest that somato-dendritic decoupling (Figures 1A,B) plays an important role in neuronal functioning, and in hierarchical cortical maturation (Figure 1C).

**Figure 2:**
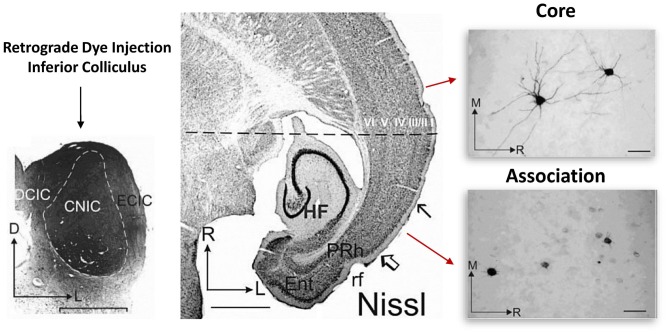
Somato-dendritic transport differences between primary and association cortical pyramidal neurons. *Left*: illustrates the experimental approach. Retrograde dye (for transport and staining) was injected into the adult inferior colliculus and staining was subsequently evaluated in both the core and association temporal lobe cortical regions. The core region represents the primary auditory cortical region. *Middle*: region between the open arrow and solid arrow indicate the association area, while the rostral cortical region (above the solid arrow) represents the core region. *Right*: single cell images from each region. Note that unlike in the association area, in the core region, somatic and dendritic labeling of single cells was much more evident. Figure adapted by permission from Bajo and Moore (2005), John Wiley and Sons Inc. © 2005 Wiley-Liss, Inc.

Earlier work focusing on primary sensory and association cortices revealed that cellular retrograde transport of dye injected into the brainstem consistently labeled deep layer cortical neuron dendrites in the adult primary sensory cortical region but not in the association cortical region of the temporal lobe (Bajo and Moore, 2005). In the association cortex, dye appeared to label only the soma (Figure 2; Bajo and Moore, 2005). This is interesting, as it indicates that something about the cellular transport mechanisms in association neurons may be quite different than those of primary sensory cortical neurons. Indeed, we have also recently reported a functional disconnection between the soma and dendrites in association cortical neurons (Chomiak et al., 2016). Importantly, this somato-dendritic decoupling was age-dependent, as the proportion of association cortical neurons exhibiting somato-dendritic decoupling decreased during the peri-adolescent period (Chomiak et al., 2016). We have proposed that a complex cytosolic lipid-protein structure may provide a mechanism of increased somato-dendritic inferfacial resistance and somato-dendritic decoupling (Chomiak et al., 2016). These findings may be related to previous electron microscopy data suggesting that endoplasmic reticulum cisternae and Nissl-stained substance appear to form a physical block or barrier that is particularly prominent at the base of dendrites and axon hillock in some pyramidal neurons (Peters and Kaiserman-Abramof, 1970).

An important question that remains unresolved is whether association cortical neurons are fundamentally different than primary sensory cortical neurons at the molecular level. It may be that the same maturational mechanisms apply to these regional cell types, although on a much slower timescale in association cortical regions. Future research will ultimately help us to better understand the mechanisms that control hierarchical cortical maturation, and whether the maturational process is initiated later or just takes longer in association cortical regions.

## Why Do Cortical Hierarchies Maintain Regional and Pathway-Specific Maturation Speeds?

At a minimum, a hierarchical mechanism of maturation allows for basic functions to stabilize, after which higher-order functions can be established. However, it is likely that there are other functions for hierarchical maturation. First, neural circuits in the mammalian brain are energetically expensive (Attwell and Laughlin, 2001; Laughlin and Sejnowski, 2003; Lennie, 2003; Harris et al., 2012). The majority of this energy expenditure is derived from re-establishing electrochemical gradients as a result of neuronal and synaptic network activity (Attwell and Laughlin, 2001; Harris et al., 2012). Given a lack of cortical neurogenesis (Rakic, 2006), a necessary consequence of hierarchical cortical maturation is that the basic circuitry hardware must be established very early during development and retained throughout postnatal development. This paradox would seem to violate the basic biological principle of efficient, economical and minimization of energy utilization of organic systems. However, by keeping association cortical neurons in a quiescent state while lower-order sensory cortical regions mature, the metabolic costs associated with functional connectivity of higher-order cortical neural circuits may be reduced (Harris et al., 2012). This idea is particularly important as neural activity is not necessarily required for the establishment of neuronal morphology and synaptic development (Kossel et al., 1997; Frotscher et al., 2000; Balasubramaniyan et al., 2004; Sigler et al., 2017). This may therefore allow the animal to direct early postnatal energy expenditures toward other basic aspects of development and survival (e.g., increasing body mass, development of skeletal musculature, etc.). This would also permit early sensory experiences to shape lower-order sensorimotor circuits while maintaining the structural integrity of the necessary circuitry needed later in life for higher-order cognitive functioning (Guillery, 2005; Knudsen et al., 2006; Chomiak and Hu, 2013). Hence, the protracted development of higher-order cognitive circuitry may have evolved as a consequence of energy being a constraint (Lotka, 1922).

A second, somewhat related possibility is perhaps best understood from a computational perspective. For example, at a basic level, the representational capacity of a network is related to the number of ways of arranging “A” active neurons in a population of “N” functional neurons (N!/[A!(N − A)!]) and is a measure of neural network performance (Laughlin, 2001). Tasks associated with cue extraction and memory retrieval may depend in part on the capacity and fidelity of the neural processing circuitry and thus the number of functional units that can contribute to N. Similar in concept to Elman’s Starting Small Hypothesis that reduced computational resources may actually facilitate language learning by allowing incremental rule learning (Elman, 1993), hierarchical cortical maturation may help developmentally limit neural processing capacity of large-scale recurrent cortical networks to facilitate normal cognitive development. Indeed, ascending and descending processing along hierarchical pathways is a fundamental organizational principle of the cortex and is thought to be important in hierarchical cognitive representations such as perception, attention, working memory and language (Guillery, 2005; Fuster, 2006; Shipp, 2007). Importantly, disruption of this hierarchical pattern of maturation may play a vital role in abnormal cognitive development and the emergence of behavioral disorders.

Finally, infantile amnesia, the inability to remember things that happened to us when we were infants (Josselyn and Frankland, 2012), may also be related to hierarchical cortical maturation (Bachevalier, 1992). For example, hippocampal-dependent remote memory has been found to correlate inversely with active neurogenesis in the hippocampus (Akers et al., 2014). That is, at least for hippocampal-dependent memories, the ability to form stable and persistent memories only emerges at relatively late developmental periods when neurogenesis declines (Josselyn and Frankland, 2012). However, for hippocampal-independent remote memory such as cued conditioning which is thought to depend on cortical association regions (Sacco and Sacchetti, 2010; Grosso et al., 2017), the neurobiological basis may require a different mechanism as the postnatal cerebral cortex lacks significant neurogenesis (Rakic, 2006). Thus, the protracted postnatal development of higher-order remote memory cortical circuits may serve this purpose.

## Outstanding Issues—How to Best Define Maturity, and Correctly Define Developmental Trajectories

The issue of how to best define neural maturity still remains an important, yet open question. Maturation indices include parameters ranging from cellular morphology, dendritic spine shape and number, intrinsic and synaptic electrophysiological characteristics (Guillery, 2005; Watson et al., 2006; Feldmeyer and Radnikow, 2009; Cheetham and Fox, 2010; Elston and Fujita, 2014; Chomiak et al., 2016), and the expression of biochemical markers (Bernier and Parent, 1998; Bourne and Rosa, 2006). However, it is still not clear which parameters are most relevant to mature neural function. One suggested approached requires defining maturity by a combination of parameters at different developmental stages (Guillery, 2005). Vascular, glial and neuronal (e.g., soma, axon, dendritic, synaptic) components are all likely to change during development. At present, a comprehensive model of how the many different cellular elements in the brain interact is still not available (e.g., Squeglia et al., 2013; Jernigan et al., 2016).

Over the past 20 years there has been a substantial increase in the number of magnetic resonance imaging (MRI)-based imaging studies characterizing cortical developmental trajectories in humans (Walhovd et al., 2016). Translational significance of these studies lies in an ability to accurately identify cortical developmental trajectories in terms of both normal development and in disease (Chomiak and Hu, 2013; Marín, 2016; Walhovd et al., 2016). Unfortunately, earlier MRI-based studies evaluating cortical developmental trajectories in children appear to be inconsistent with more recent MRI-based studies (Walhovd et al., 2016). Earlier studies suggested that cortical thickness increases throughout the preschool years and into the school-age years, while more recent studies have suggested an opposing view, one of monotonic cortical thinning (Walhovd et al., 2016). These discrepant findings may be related to a number of factors including differences in methodology and analysis tools (Walhovd et al., 2016). Nevertheless, what is clear is that despite the inconsistencies in the developmental trajectories of cortical maturation between studies, sequential maturation such as a sensory-to-association cortical maturational gradient is a point on which these studies often agree on (Walhovd et al., 2016).

## Conclusion

In conclusion, the evidence for hierarchical cortical maturation in humans appears to be quite robust, and there is directly related evidence from animal research to support hierarchical cortical maturation (Jacobson, 1963; Bernier and Parent, 1998; Bourne and Rosa, 2006; Hishida et al., 2007; Sia and Bourne, 2008; Shi et al., 2013; Chomiak et al., 2016). The precise mechanisms that are responsible for this hierarchical cortical maturation, however, remain to be established.

## Author Contributions

TC planned and wrote the manuscript and BH critically evaluated it. Both authors contributed to the ideas related to the content.

## Conflict of Interest Statement

The authors declare that the research was conducted in the absence of any commercial or financial relationships that could be construed as a potential conflict of interest.
